# From childhood trauma to hyperarousal in adults: The mediating effect of maladaptive shame coping and insomnia

**DOI:** 10.3389/fnhum.2023.990581

**Published:** 2023-02-16

**Authors:** Frans Schalkwijk, Eus J. W. Van Someren, Nelleke J. Nicolai, Julia L. Uijttewaal, Rick Wassing

**Affiliations:** ^1^Department of Forensic Special Education, University of Amsterdam, Amsterdam, Netherlands; ^2^Netherlands Institute for Neuroscience, Amsterdam, Netherlands; ^3^Department of Integrative Neurophysiology, Center for Neurogenomics and Cognitive Research, Vrije Universiteit Amsterdam, Amsterdam, Netherlands; ^4^Private Practice, Rotterdam, Netherlands; ^5^Woolcock Institute of Medical Research, Sydney, NSW, Australia

**Keywords:** insomnia, adverse childhood experiences (ACEs), childhood trauma, hyperarousal, dorsal anterior cingulate cortex (dACC), shame

## Abstract

**Introduction:**

A new line of insomnia research focuses on the developmental trajectories from early live stress to insomnia in adulthood. Adverse childhood experiences (ACE’s) might create a vulnerability for later maladaptive coping with distress, as seen in chronic hyperarousal or insomnia. In an functional magnetic resonance imaging (fMRI) study, failure to dissociate the neurobiological components of shame from autobiographical shameful memories in insomnia was reflected by continued activation of the dorsal anterior cingulate cortex (dACC), which may be a result of maladaptive coping in the wake of ACE’s. Following up on that study, the current pilot study explores the relation between ACE’s, shame coping-styles, adult insomnia, hyperarousal, and neurobiology of autobiographical memory.

**Methods:**

We used existing data (*N* = 57) from individuals with insomnia (*N* = 27) and controls (*N* = 30), and asked these participants to complete the childhood trauma questionnaire (CTQ). Two structural equation models were used to test the hypotheses that shame-coping styles and insomnia symptom severity mediate the association between ACE’s and (1) self-rated hyperarousal symptoms and (2) dACC activation to recall of autobiographical memories.

**Results:**

For the association between ACE’s and hyperarousal, there was a significant mediation of shame-coping style (*p* < 0.05). This model also indicated worse shame coping with more ACE’s (*p* < 0.05) and worse insomnia symptoms with more ACES’s (*p* < 0.05), but no association between shame coping and insomnia symptoms (*p* = 0.154). In contrast, dACC activation to recall of autobiographical memories could only be explained by its direct association with ACE’s (*p* < 0.05), albeit that in this model more ACE’s were also associated with worse insomnia symptoms.

**Discussion:**

These findings could have an implication for the approach of treatment for insomnia. It could be focused more on trauma and emotional processing instead of conventional sleep interventions. Future studies are recommended to investigate the relationship mechanism between childhood trauma and insomnia, with additional factors of attachment styles, personality, and temperament.

## 1. Introduction

Adverse childhood experiences (ACEs) have a devastating effect on adult emotional functioning, especially if the ACEs are multiple, leading to higher risks for depressive illness, anxiety, addiction, suicidality, and medical illness (DeBellis, 2010). ACEs are negative experiences, such as emotional abuse or neglect, physical abuse or neglect, sexual abuse, and exposure to parental substance abuse, mental illness, or criminal behavior (Felitti et al., 1998). Other than traumatic situations, such as a car accident, a landslide, or war, ACEs are often inflicted by the caretakers, who in their position were expected to protect and regulate the emotions of the child. In psychoanalytic practice, we see patients with ACEs struggling with a chronic state of hyperarousal (Van der Kolk, 2014) and relationship anxiety (Morrison, 1989; Dorahy et al., 2016, 2017). Their self- and emotion-regulation strategies are limited, as they have internalized dysregulating caretakers that have been a threat to self-cohesion (DeYoung, 2015). Hyperarousal causes a collapse of reflective functioning; emotion and affects are no longer experienced as temporary (conscious and verbal) feeling states but as states reflecting identity (Fonagy et al., 2002). Thus, feelings, such as guilt and shame are experienced as “I am a bad person,” which reflects an equivalent mode of functioning (Allen et al., 2006).

Hyperarousal can be described not only by the above psychological factors but also by the physiological processes involved in arousal, including increased high-frequency electroencephalography (EEG) activity, hormone secretion (e.g., cortisol), limbic brain metabolism, and sympathetic activation (Riemann et al., 2010). Importantly, hyperarousal is considered one of the primary risk factors for the development of mental health problems.

In a series of papers reviewed below, we studied the relationship between hyperarousal and shameful memories from the past in patients suffering from insomnia. Following up on these papers, in the research presented here, we aim to further explain hyperarousal and shame coping by examining more severe negative experiences faced in childhood. We studied the mediating effect of shame coping and insomnia on ACEs and hyperarousal.

A first factor that links ACEs to hyperarousal may be maladaptive shame coping styles. Clinically, a distinction is made between adaptive and maladaptive shame. The former refers to bypassing feelings of shame, which contribute to adaptive self-esteem regulation and social communication (Schalkwijk et al., 2016). The latter is characterized by maladaptive thoughts or behaviors referring to negative self-esteem, and a desire to hide or escape, and is experienced as a negative feeling threatening identity (Luyten et al., 2002). ACEs negatively influence the development of a child’s identity. Often, the sense of self is threatened and the child questions his or her role in these experiences, such as “Who am I, when they can do this to me?” (Lewis, 1971; Tracy et al., 2007; Lichtenberg et al., 2011; Schalkwijk, 2012). Usually, the personality of persons with many ACEs is characterized by feelings of shame, powerlessness, and lack of a sense of agency (Lanius et al., 2020). When circumstances are chronically unfavorable, as in the case of many ACEs, a child’s personality is characterized by maladaptive shame-coping strategies. The shame manifests either as blame for their shortcomings (internalizing) or attributing blame to others (externalizing), which can lead to hyperarousal (Wassing et al., 2016) and mental health disorders (Campbell, 2016).

A second factor linking ACEs to hyperarousal is insomnia (Bader et al., 2007), which is a sleep disorder characterized by hyperarousal (Riemann et al., 2010) and is in itself a primary risk factor for the development of mental health problems (Manber et al., 2008; Baglioni et al., 2011; Christensen et al., 2016; Blom et al., 2017; Hertenstein et al., 2019). Bader et al. (2007) found that 46% of the participants with insomnia reported moderate to severe ACEs, and the number of ACEs has been found to be proportional to the severity of insomnia (Wang et al., 2016). Furthermore, Kajeepeta et al. (2015) found evidence of an association between witnessing family conflict and sexual abuse, and insomnia. Moreover, more ACEs have been linked to disturbed rapid eye movement (REM) sleep in adulthood (Insana et al., 2012), which is a consistent marker not only of insomnia but also of other mental health disorders (Germain et al., 2008; Riemann et al., 2012; Pesonen et al., 2019).

Concerning these possible mediation effects between ACEs and hyperarousal, genome-wide association studies followed up by functional annotation analysis suggest strong correlations between the expression of insomnia risk genes, psychiatric traits, and the limbic circuitry (Jansen et al., 2019). Furthermore, a recent systematic review presented consistent findings of altered epigenetic modifications of the glucocorticoid gene NRC31 in adults exposed to ACEs (Lang et al., 2020), which is in line with persistent changes in the function of the hypothalamic-pituitary axis observed in mental health disorders (Griffiths and Hunter, 2014). Importantly, patients with insomnia do not necessarily have increased emotional reactivity to novel experiences, i.e., they do not show stronger initial emotional responses. A recent study with 25,758 participants of the United Kingdom Biobank showed no association between insomnia symptoms and limbic brain activation during an emotional task (Schiel et al., 2022). However, the differences from regular sleepers become evident after sleeping (Wassing and D’Rozario, 2022). Following a night with disturbed REM sleep, patients with insomnia show continued emotional reactivity, which may last for months or even years, and could explain a chronic hyperarousal state (Wassing et al., 2016). It has been proposed that adaptive emotion regulation depends on sound REM sleep (Walker, 2009; Walker and Van der Helm, 2009). During REM sleep, a selective memory consolidation process may support the disengagement of the emotional limbic and salience brain networks. Wassing et al. (2019a) found support for the hypothesis that maladaptive sleep in insomnia perturbs overnight emotion regulation and may contribute to chronic hyperarousal (Wassing et al., 2016). Wassing et al. (2019b) also found support for the hypothesis that chronic hyperarousal in insomnia disorder is associated with the failure to dissociate the limbic circuitry from long-term memory traces. Patients with insomnia who recalled autobiographical shameful memories showed stronger blood-oxygen-level-dependent (BOLD) responses in the dorsal anterior cingulate cortex (dACC), which is part of the limbic and salience circuitry. Recall of adverse emotional memories also activates the dACC in patients with post-traumatic stress disorder (PTSD; Whalley et al., 2013). PTSD can be conceptualized as a disorder of impaired fear-extinction memory, and delayed acquisition of the fear-extinction memory is linked with continued activation of the dACC (Seo et al., 2018). The dACC, otherwise known as the mid-cingulate cortex, has predominant connections with the amygdala, striatum, and dopaminergic and serotonergic systems, and is functionally linked with evaluating prediction errors, updating memory schema, and adapting/persisting behavioral responses (Kolling et al., 2016; van Heukelum et al., 2020). Considering these findings, we aimed to identify the dACC as the neurobiological expression of hyperarousal. Since the dACC is most consistently shown to be involved in emotional memory tasks in insomnia and PTSD populations (Whalley et al., 2013; Seo et al., 2018; Wassing et al., 2019b) we limited our analyses to the dACC and not to other nodes of the salience or limbic network.

Following this line of research, in this exploratory study, we asked ourselves where the vulnerability for hyperarousal and aberrant neurobiology of autobiographical memory in people with insomnia might originate. Could ACEs lead to the vulnerability of insomnia by weakening the stress regulatory processes?

To the best of our knowledge, this is the first exploratory study that investigated the relationship between ACEs, maladaptive shame coping, insomnia, hyperarousal, and dACC activation in the recall of autobiographical memories. In order to evaluate these relationships, we employed structural equation modeling with one direct and two parallel mediation paths so that all covariance between the variables of the hypothesized paths could be fitted to the observed data at once. We used two structural equation models to test the hypotheses that shame-coping styles and insomnia symptom severity mediate the association between ACEs and (1) self-rated hyperarousal symptoms, and (2) dACC activation in recalling autobiographical memories. Furthermore, we expected that more ACEs would result in worse maladaptive shame-coping styles and insomnia severity.

## 2. Materials and methods

### 2.1. Participants

The 57 participants in this study were described by Wassing et al. (2019b). These participants included 27 patients that fulfilled the diagnostic criteria for insomnia disorder [mean, standard deviation (SD) age, 45.5 (13.4) years; 10 men; ISI range 8–26] and regular sleepers [*n* = 30; mean [SD] age, 42.4 (15.8) years; 17 men, ISI range 0–14]. The participants answered the self-report questionnaires approximately 1 week before the functional magnetic resonance imaging (fMRI) scan.

Since the childhood trauma Questionnaire (CTQ) (Bernstein et al., 2003) was not part of this dataset established in 2015–2016, in February 2021 we asked these participants to complete the CTQ, and 44 participants responded (77%, *n* = 23 patients with insomnia, *n* = 21 regular sleepers).

This study was approved by the Ethical Committee of the Department of Education of the University of Amsterdam (approval number 3421). Written informed consent was obtained from the participants prior to enrollment in the study.

### 2.2. Measures

#### 2.2.1. ACEs

Adverse childhood experiences were assessed using the Dutch version of the CTQ (Bernstein et al., 1997; Thombs et al., 2009; Spinhoven et al., 2014). It consists of 25 items rated on a five-point Likert scale (1 = never true, 5 = very often true) and measures five categories of trauma: emotional abuse, physical abuse, sexual abuse, physical neglect, and emotional neglect. An example of a physical neglect item is “During my childhood… I had to wear dirty clothes.” The CTQ demonstrated a Cronbach’s alpha of 0.95 for the total scale. The Cronbach’s alpha for the subscales were 0.91 for physical abuse, 0.89 for emotional abuse, 0.95 for sexual abuse, 0.63 for physical neglect, and 0.91 for emotional neglect.

#### 2.2.2. Coping with shame

Coping with shame was assessed using the Compass of Shame Scale (CoSS) (Elison et al., 2006; Schalkwijk et al., 2016). This self-report questionnaire consists of 15 shame-inducing situations and six answer categories. The participants were asked about their reaction to a situation, such as “When an activity makes me feel like my strength or skill is inferior,” and a reaction, such as “I get mad at myself for not being good enough,” on a five-point Likert scale (1 = never, 5 = almost always). This scale measures two maladaptive shame-coping styles: internalizing and externalizing. Internalizing shame coping contains answers in which the person blames himself or herself for being ashamed (“I brood over my flaws”) or the person avoids the shame-provoking situation (“I want to disappear”). Externalizing shame coping includes items in which the person blames others for feeling shame (“I get irritated with other people”) or denies that the situation evokes shame (“I act as if it isn’t so”). Two other categories assess adaptive shame coping (“I think, that was stupid of me, but that’s what happens to everyone”) and proneness to experience shame (“I am ashamed”). The Cronbach’s alpha for the subscales were as follows: avoidance, 0.75; attack other, 0.76; attack self, 0.86; withdrawal, 0.75; adaptive, 0.78; and shame proneness, 0.87.

#### 2.2.3. Insomnia

Insomnia symptom severity was assessed using the Insomnia Severity Index (ISI), a self-report measure used to assess the nature, severity, and impact of insomnia (Bastien et al., 2001). A five-point Likert scale was used (0 = no problem, 4 = severe problem), with a total score ranging from 0 to 28. An example item is “Please rate the current SEVERITY of your insomnia problems: difficulty falling asleep.” The ISI demonstrated a Cronbach’s alpha of 0.76.

#### 2.2.4. Hyperarousal

Hyperarousal was assessed using the Hyperarousal Scale (HS; Pavlova et al., 2001), a self-report questionnaire consisting of 26 items in the form of statements. Participants judged the statements using four different responses (0 = not at all, 1 = a little, 2 = quite a bit, and 3 = extremely). An example item is “I keep thinking about things long after they happened.” The HS demonstrated a Cronbach’s alpha of 0.81 (Bruno et al., 2020). Note that two out of the 26 items overlap with those in the ISI: “I am slow to awaken in the mornings” (HS) may be anticorrelated with “problems waking up too early” (ISI), and “I have trouble falling asleep” (HS) is likely to be correlated with “difficulty falling asleep” (ISI).

#### 2.2.5. dACC reactivity to recall of autobiographical memories

The dACC BOLD responses to shameful experiences were obtained during fMRI recordings. For a detailed description of the procedures, functional and structural MRI acquisition, data processing, and statistical analysis see Wassing et al. (2019b). In summary, 1 week prior to the fMRI scan, participants were asked to think of up to ten shameful past experiences, and for each to write down four cue words that, when prompted, allowed them to recall and relive the experience. In addition, for each shameful experience, participants were instructed to also write four cue words for neutral events from the same period (e.g., traveling to work/school). During the fMRI scan, the cue words of each of the experiences were presented on the screen for 16 s. A total of 10 blocks of shameful conditions and 10 blocks of neutral conditions were presented in pseudorandom order. The raw fMRI data were preprocessed using FMRIB Software Library (FSL)^1^ including corrections for inhomogeneities in the B0-field, motion correction using registration and independent component analysis with automatic removal of motion artifacts (ICA-AROMA) (Pruim et al., 2015), spatial smoothing (5 mm full width at half maximum kernel), grand-mean intensity normalization, nuisance signal regression (from cerebrospinal fluid and white-matter brain masks), and temporal high-pass filtering (1/90 Hz). The mean dACC activation for each participant was obtained using linear regression models in FSL FMRI Expert Analysis Tool (FEAT) (Woolrich et al., 2001) including a contrast on the regressors (shameful vs. neutral) and a mask of the dACC obtained in Wassing et al. (2019b).

### 2.3. Statistical approach

The hypotheses were tested using structural equation modeling in R (version 4.1.2, R Foundation, Vienna, Austria) and RStudio (2022.02.3 Build 492) using the *lavaan* package (R Core Team, 2021). Structural equation modeling is a statistical approach that combines confirmatory factor analysis to extract latent variables from observed variables with regression analysis to estimate and evaluate the statistical significance of hypothesized paths. Instead of performing separate linear models per path, the technique uses the multivariate covariance structure of the observed data to fit the model all at once. We employed two models, one with self-rated hyperarousal as the outcome measure, and one for dACC reactivity to recall autobiographic memories. Path coefficients were obtained using maximum likelihood estimators and considered significant at a two-sided alpha threshold of 0.05. Finally, full mediation was indicated by a significant mediation path estimate in combination with a non-significant direct path estimate between ACES and the outcome variable. A partial mediation was indicated if both the direct path estimate and the mediation path estimate were significant.

#### 2.3.1. ACEs and self-rated hyperarousal

The measurement model included four latent variables: (1) “ACES” were measured by the observed scores on the CTQ subscales of emotional neglect, physical neglect, emotional abuse, physical abuse, and sexual abuse; (2) “shame” was measured by the CoSS subscales of shame-proneness, adaptive shame coping, and internalizing and externalizing maladaptive coping; (3) “insomnia” was directly measured by the ISI total score; and (4) “hyperarousal” was directly measured by the hyperarousal-scale total score. The regression model included three equations, all of which included age and sex as confounders. First, shame was predicted by ACEs. Second, insomnia was predicted by ACEs and shame. Third, hyperarousal was predicted by ACES, shame, and insomnia scores. No moderating effects were considered. Mediation path estimates were computed where shame, insomnia, and both shame and insomnia mediated the association between ACEs and hyperarousal.

#### 2.3.2. ACES and dACC reactivity to recall autobiographical memories

This structural equation model was similar to the one described in the previous subsection, except that the “hyperarousal” latent variable was replaced with the dACC BOLD responses for relived shameful experiences. Note that the path estimates in the two models between the same variables (e.g., ACEs to insomnia) could be different because the variance explained in ACEs by hyperarousal (model 1) was different from its variance explained by dACC BOLD responses (model 2). This means that the remaining variance in ACEs that was *not* explained by the outcome variable but *was* explained by insomnia could be different too.

## 3. Results

### 3.1. Descriptive ACEs statistics

The mean and SD values of the five CTQ subscales are shown in Table 1. Each subscale contained five question items, and their scores ranged from 5 to 25. Sexual abuse had the lowest mean score (*M* = 5.27), close to the lowest possible score. Emotional neglect had the highest mean score (*M* = 12.16), which was half of the highest possible subscale score.

**TABLE 1:** Descriptive statistics of adverse childhood experiences (ACEs).

	Mean	SD	Range
Emotional neglect	12.16	5.55	5–25
Physical neglect	7.05	2.16	5–12
Emotional abuse	8.68	4.20	5–20
Physical abuse	6.33	3.04	5–20
Sexual abuse	5.27	0.90	5–10

### 3.2. Correlation structure among observed variables

**FIGURE 1:**
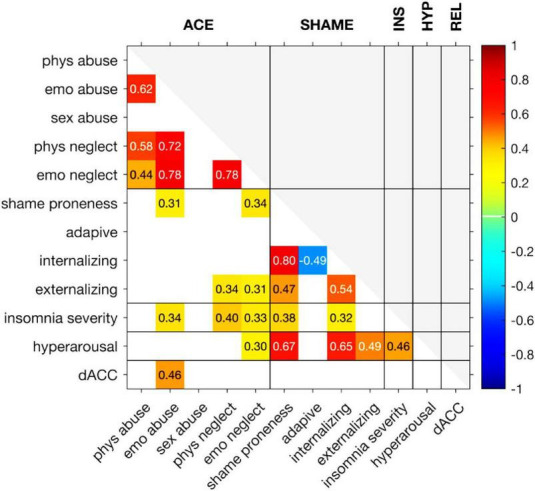
Correlation between observed variables. The color indicates the strength of the correlation. Only correlations with *p*-value below 0.05 are shown. Abbreviations from left upwards to left downwards: physical abuse, emotional abuse, sexual abuse, physical neglect, emotional neglect, shame proneness, adaptive shame coping, internalizing shame coping, externalizing shame coping, insomnia severity, hyperarousal, and dorsal anterior cingulate cortex. Abbreviations from left upward to right upward: adverse childhood experiences (ACEs), Shame, Insomnia Severity (INS), Hyperarousal (HYP), reliving shameful experiences (REL).

The correlations between observed variables are shown in Figure 1. Concerning the CTQ subscales, the ISI total score was correlated with emotional abuse (*r* = 0.34, *p* < 0.03), physical neglect (*r* = 0.40, *p* < 0.007), and emotional neglect (*r* = 0.33, *p* < 0.03). Emotional abuse was also correlated with shame proneness (*r* = 0.31, *p* < 0.04), physical neglect with maladaptive externalizing shame coping (*r* = 0.34, *p* < 0.03), and emotional neglect was correlated with both shame proneness (*r* = 0.34, *p* < 0.03) and maladaptive externalizing shame coping (*r* = 0.31, *p* < 0.05). Most CTQ subscales were not associated with hyperarousal or dACC responses to reliving shameful experiences. Only emotional neglect correlated significantly with hyperarousal (*r* = 0.30, *p* < 0.05), and emotional abuse with dACC responses to reliving shameful experiences (*r* = 0.46, *p* < 0.002).

Concerning the CoSS subscales, shame proneness was correlated with the ISI total score (*r* = 0.38, *p* < 0.02) and hyperarousal (*r* = 0.67, *p* < 10^–6^). In addition, maladaptive internalizing shame coping was correlated with the ISI total score (*r* = 0.32, *p* < 0.04) and hyperarousal (*r* = 0.65, *p* < 10^–5^). Finally, maladaptive externalizing shame coping was correlated with hyperarousal (*r* = 0.49, *p* < 0.001).

The ISI total score was correlated with hyperarousal (*r* = 0.46, *p* < 0.002).

### 3.3. Structural equation modeling

#### 3.3.1. ACEs and hyperarousal

**FIGURE 2:**
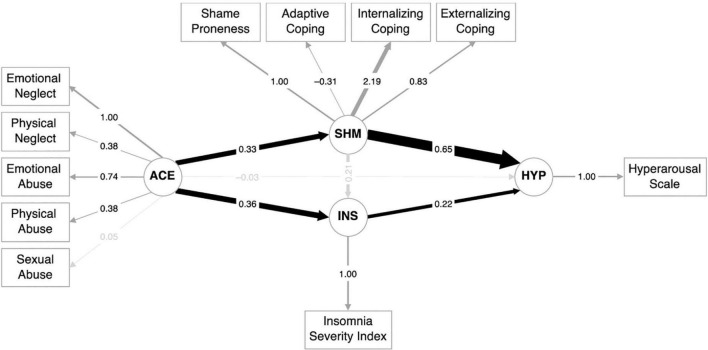
Structural equation model for hyperarousal.

All the model-fit indices combined indicated a satisfactory to a good fit. The Root Mean Square Error of Approximation (RMSEA) was 0.09 (*p* = 0.14; the null hypothesis that the RMSEA was less than or equal to 0.05, could not be rejected, indicating a good fit). However, the discrepancy between the observed and fitted covariance matrices was barely significant (χ^2^(56) = 74.7, *p* = 0.048). The sample-size insensitive metrics also indicated a satisfactory to a good fit, that is, the comparative fit index = 0.92 (good) and the Tucker–Lewis index = 0.89 (satisfactory > 0.90). The factor loadings of the observed variables on the latent variables are shown in Figure 2. Of note, sexual abuse was loaded onto the ACES latent variable with trend-level significance (*p* = 0.09). The structural equation model for hyperarousal is shown in Figure 2.

#### 3.3.2. Path estimates

Adverse childhood experiences did not directly predict hyperarousal (*p* = 0.98), but there were significant associations between ACEs and shame [β (SD) = 0.05 (0.02), standardized β = 0.33, *Z* = 2.08, *p* < 0.04] and ACEs and insomnia [β (SD) = 0.58 (0.24), standardized β = 0.36, *Z* = 2.42, *p* < 0.02]. However, insomnia was not predicted by shame (*p* = 0.17). Hyperarousal was predicted by shame [β (SD) = 7.30 (1.42), standardized β = 0.65, *Z* = 5.15, *p* < 10^–6^] and insomnia [β (SD) = 0.21 (0.11), standardized β = 0.22, *Z* = 1.96, *p* < 0.05]. The combined path estimate from ACEs *via* insomnia to hyperarousal was not significant [β (SD) = 0.12 (0.08), standardized β = 0.08, *Z* = 1.50, *p* = 0.13]. Finally, the combined path estimate from ACEs *via* shame to hyperarousal was significant [β (SD) = 0.33 (0.17), standardized β = 0.21, *Z* = 1.98, *p* < 0.05], indicating that the association between ACEs and hyperarousal was fully mediated by shame.

### 3.4. ACEs and dACC reactivity to recall of autobiographical memories

All model-fit indices combined indicated a good fit. The RMSEA was 0.06 (*p* = 0.39), and there was no significant discrepancy between the observed and fitted covariance matrices (χ^2^(56) = 64.9, *p* = 0.20). The comparative fit index (0.95) and Tucker–Lewis index (0.94) also indicated a good fit.

#### 3.4.1. Path estimates

**FIGURE 3:**
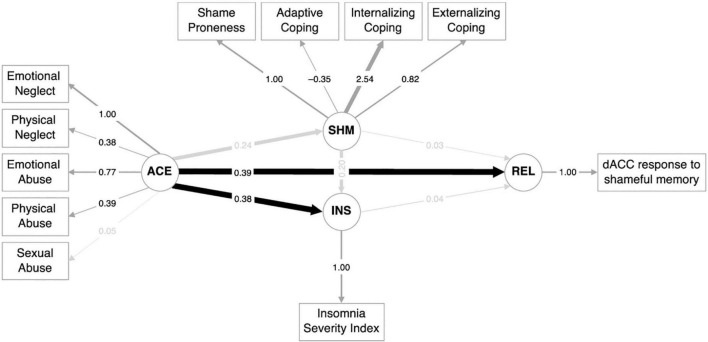
Structural equation model for dorsal anterior cingulate cortex (dACC) reactivity to recall of autobiographical memories.

Adverse childhood experiences directly predicted the dACC responses to relived experiences [β (SD) = 0.07 (0.03), standardized β = 0.39, *Z* = 2.38, *p* < 0.02]. Furthermore, while ACEs did not predict shame in this model (*p* = 0.12), ACES did predict insomnia [β (SD) = 0.62 (0.24), standardized β = 0.38, *Z* = 2.62, *p* < 0.01], although insomnia was not predicted by shame (*p* = 0.15). Finally, there were no associations between dACC responses to relived experiences and shame (*p* = 0.85) or insomnia (*p* = 0.82), which also indicated that no mediation paths were significant (*p* > 0.82). The structural equation model for dACC reactivity to recall autobiographical memories is shown in Figure 3.

## 4. Discussion

This exploratory study investigated the mediating effect of dysfunctional shame regulation strategies and insomnia between ACES and emotionally experienced chronic hyperarousal, and its potential neurobiological underpinnings.

Our main interest in this study was the mediating effect of dysfunctional shame regulation strategies and insomnia between ACEs and emotionally experienced chronic hyperarousal and dACC responses to reliving shameful experiences. The bivariate correlation structure showed that, from the five groups of ACEs, emotional neglect seemed to have the most impact on hyperarousal and emotional abuse on dACC responses to reliving shameful experiences. Indeed, emotional abuse and neglect have been linked with the most long-term detrimental effects (Strathearn et al., 2020). Clinically speaking, maladaptive shame regulation strategies have been developed. Note that in this first structural equation model, insomnia did not significantly mediate the association between ACEs and hyperarousal, whereas indeed a significant mediation effect was found for maladaptive shame coping styles.

In the second structural equation model, our interest was the mediating effect of dysfunctional shame regulation strategies and insomnia between ACEs and the dACC response to reliving shameful memories. In contrast to the first model, we found no mediational effects for maladaptive shame regulation, nor for insomnia symptom severity. In a study by Wassing et al. (2019b), the insomnia patients, but not the controls, demonstrated dACC responses to the shame-inducing recall of autobiographical memories. In the current study, we now show that more ACEs relate to more severe insomnia symptoms and that insomnia severity did not mediate the association between ACEs and dACC responses. This may indicate that ACEs independently contribute to dACC responses when recalling shameful experiences. According to Van der Kolk’s (2014) book title, the body does keep score of these events leading to hyperarousal. Our findings support the importance of explicitly addressing the component of shame in ACEs during treatment.

Secondly, we discuss the bivariate correlation structure reported in Figure 1. Here we analyzed the premise that more ACEs lead to increased insomnia (Bader et al., 2007; Wang et al., 2016; Muscatello et al., 2020; Reffi et al., 2022). This was confirmed: having experienced more ACEs was predictive of insomnia severity in adults. However, this was found merely for the ACEs of emotional neglect, emotional abuse, and physical neglect. Differently from what happens with ACEs that include physical intrusions, such as sexual or physical abuse, other types of ACEs, such as emotional neglect, emotional abuse, and physical neglect, may go unnoticed as a child may never address or voice these experiences. In these cases, children tend to react physically and emotionally to these experiences with maladaptive coping strategies. Prior work has shown that emotional abuse and neglect can have the most detrimental effects long term (DeBellis, 2010). These kinds of ACEs are associated with much more adverse outcomes in areas of cognition and education, psychological and mental health, addiction and substance use, and sexual and physical health than the physically intrusive ACEs (Strathearn et al., 2020). In our study, we showed the association between maladaptive shame regulation strategies and experiencing a subjective state of chronic hyperarousal (Schore, 2003; Porges, 2011). Sexual and physical abuse, however, is associated with the violation of body integrity, which is a far more obvious violation for a child to notice and these might lead to other coping strategies. A typical outcome of sexual abuse is lifelong PTSD and depression (Strathearn et al., 2020), dissociation (Draijer and Langeland, 1999; Dorahy et al., 2017; Vonderlin et al., 2018; Nicolai, 2020; Voestermans et al., 2021), and self-destructive behavior. Dissociation protects the individual from experiencing the painful emotions experienced during the abuse, such as physical pain, shame, disgust, and helplessness. Also, there is no sexual abuse without neglect (Draijer, 1990). In other words, ACEs seldom take place alone.

This pilot study has several limitations. The number of participants was small and they were recruited based on insomnia rather than trauma. Therefore, those who were physically and sexually abused probably were underrepresented in our sample and the distribution of male and female participants was uneven. Another limitation was caused by the psychological effect of chronic arousal mentioned in the introduction. We have discussed that chronic hyperarousal hampers reflective functioning and people have limited capacity for recognizing (conscious and verbal) feeling states. Feelings cannot be differentiated or reflected upon, so feelings of shame, guilt, and anxiety end up confirming the cognition of preconceived notions, such as that the individual who suffered ACESs is bad, stupid, and disgusting. The found effect of ACEs on chronic hyperarousal might thus be underreported, as people have difficulties in reflecting on their (conscious and verbal) feeling states.

A few recommendations are suggested from this study. A larger sample from a variety of clinical institutions would better represent a population with insomnia. In addition, future research should consider that adult survivors of childhood abuse have different trajectories leading to insomnia (Steine et al., 2019). For example, another interesting mediating variable might be attachment style, as research has shown that shame and hyperarousal are associated implicitly with attachment styles, the way people engage in close and intimate relationships (Van der Kolk, 2014). Sedighimornani et al. (2021) found that shame was positively and significantly associated with insecure, fearful, and anxious attachment styles, whereas individuals with a secure attachment style had lower levels of shame (Gross and Hansen, 2000). A clinical example of the relationship between shame and unresolved and entangled attachment is found in borderline personality disorder (BPD). BPD is characterized by higher levels of baseline emotional intensity for guilt, shame, and fear, and higher levels of shame reactivity (Di Bartolomeo et al., 2022; Estric and Lopez-Castroman, 2022). A “chicken-and-egg” discussion is still ongoing concerning the relationship between attachment and temperament, another possible mediating variable (Hong and Park, 2012). Concerning the Big Five personality traits, e.g., high levels of neuroticism are associated with high levels of experiencing shame, whereas high levels of extraversion are associated with less shame (Erden and Akbag, 2015). Palagini et al. (2018) found that, in turn, insecure attachment in patients with insomnia has been associated with hyperarousal, pre-sleep hyperarousal, and emotion dysregulation. Therefore, clinically, we would expect shame and insomnia of having a mediating role between ACES and insecure attachment.

## Data availability statement

The raw data supporting the conclusions of this article will be made available by the authors, without undue reservation.

## Ethics statement

The studies involving human participants were reviewed and approved by the Universiteit van Amsterdam. The patients/participants provided their written informed consent to participate in this study.

## Author contributions

FS, EV, and RW contributed equally to this research. JU participated as a graduate student. NN as a clinical expert. All authors contributed to the article and approved the submitted version.

## Footnotes

1
https://fsl.fmrib.ox.ac.uk
